# Report from the 4th European Bone Sarcoma Networking meeting: focus on osteosarcoma

**DOI:** 10.1186/s13569-018-0103-0

**Published:** 2018-08-10

**Authors:** Sandra J. Strauss, Jakob Anninga, Rubina Baglio, Daniel Baumhoer, Sam Behjati, Stefan Bielack, Kjetil Boye, Javier M. Broto, Anne-Marie Cleton-Jansen, Andrea Degasperi, Abigail Evans, Franca Fagioli, Marta Fiocco, Nathalie Gaspar, Dominique Heymann, Nadia Hindi, Carlo Lancia, Ola Myklebost, Michaela Nathrath, Francoise Redini, Katia Scotlandi, Elisa Tirtei, Michel Vanden Eynden, Jeremy Whelan

**Affiliations:** 1 Department of Oncology, UCL Cancer Institute, 72 Huntley Street, London, WC1A 6DD UK; 2 University College London Hospitals, London, UK; 3 Radboud University Medical Center, Nijmegen, The Netherlands; 4 Cancer Center Amsterdam, VU University Medical Center, Amsterdam, The Netherlands; 5 Bone Tumour Reference Centre at the Institute of Pathology, University Hospital Basel, Basel, Switzerland; 6 Wellcome Trust Sanger Institute, Cambridge, UK; 7 Universitätsklinikum Münster, Münster, Germany; 8 Klinikum Stuttgart-Olgahospital, Stuttgart, Germany; 9 Oslo University Hospital, Oslo, Norway; 10 Hospital Universitario Virgen del Rocío, Seville, Spain; 11 Leiden University Medical Center, Leiden, The Netherlands; 12 University of Cambridge, Cambridge, UK; 13 Regina Margherita Children’s Hospital, A.O.U. Città della Salute e della Scienza, Turin, Italy; 14 Leiden University, Leiden, The Netherlands; 15 Gustave-Roussy Cancer Campus, Villejuif, France; 16 Sarcoma Research Unit, EAL, INSERM, University of Sheffield, Sheffield, UK; 17 University of Bergen, Bergen, Norway; 18 Technische Universität München, Munich, Germany; 19 Klinikum Kassel, Kassel, Germany; 20 UMR1238 INSERM UnivNantes, Nantes, France; 21 Istituto Ortopedico Rizzoli, Bologna, Italy; 22 Cliniques Universitaires Saint-Luc, Brussels, Belgium; 23 Université Catholique de Louvain, Brussels, Belgium

**Keywords:** Osteosarcoma, Bone sarcoma, Immunotherapy, Genomics, Translational research

## Abstract

This report summarizes the proceedings of the 4th European Bone Sarcoma Networking Meeting, held in London, England, on 21 June 2017. The meeting brought together scientific and clinical researchers and representatives from sarcoma charities from 19 countries representing five networks across Europe, to present and discuss new developments on bone sarcoma. In view of the challenges is poses, the meeting focussed primarily on osteosarcoma with presentations on developments in our understanding of osteosarcoma genetics and immunology as well as results from preclinical investigations and discussion of recent and ongoing clinical trials. These include studies examining the efficacy of multi-targeted tyrosine kinase inhibitors and checkpoint inhibitors, as well as those with molecular profiling to stratify patients for specific therapies. Discussion was centred on generation of new hypotheses for collaborative biological and clinical investigations, the ultimate goal being to improve therapy and outcome in patients with bone sarcomas.

## Introduction

The 4th European Bone Sarcoma Networking meeting brought together scientific and clinical researchers and representatives from sarcoma charities, from 19 countries representing five networks across Europe to discuss the latest developments in bone sarcoma research. This builds on the successful previous meetings [1]. Since the closure of the EURAMOS-1 trial in 2011 there have been no international phase III studies open for European patients with osteosarcoma. In view of this ongoing unmet need, the meeting focussed primarily on this challenging disease. The meeting opened with presentations from National Study Groups on current clinical trials as well as those in development, including a number of immunotherapy-based trials. Potential therapies based on recent osteosarcoma genetic studies and subsequently immunology and immunotherapy were discussed as well as the role for biomarkers both to identify patients for these studies and for monitoring of disease.

## Current national study programmes

The first session was an opportunity for national study groups to present recent clinical trials that have recruited patients with osteosarcoma, are currently recruiting osteosarcoma patients or are under development (summarised in Table 1). The Spanish Group for Research on Sarcoma (GEIS) represented by Nadia Hindi, presented data from GEIS 29 trial, a phase II trial on the combination of gemcitabine and sirolimus [2]. In this study, patients with advanced pretreated osteosarcoma received up to six cycles of gemcitabine 800 mg/m^2^ days 1 and 8 and Sirolimus (5 mg per day) in a continuous regimen (except the day before and the day of infusion of gemcitabine). The trial was positive for its primary endpoint, with a 4-month PFS rate of 44%. Median PFS and OS were 2.3 and 7.1 months respectively. Expression of Ribonucleotide Reductase catalytic subunit 1 (RRM1) was related with a worse outcome, both in terms of PFS and OS [2]. The GEIS group has another two phase II trials actively recruiting patients with advanced osteosarcoma: GEIS 51 (Palbosarc), which is examining the role of the CDK4 inhibitor palbociclib in patients with osteosarcoma or soft tissue sarcoma overexpressing CDK4 and GEIS 52 (InmunoSARC), which is examining the tolerance and efficacy of the combination of sunitinib and nivolumab in patients with bone and soft tissue sarcomas [3, 4].

**Table 1:** Recent European clinical trials recruiting patients with osteosarcoma

Trial name	Title/description	Country	Study reference
GEIS 29	Multicenter and prospective phase II trial with gemcitabine and rapamycin in second line of metastatic osteosarcoma	Spain	NCT02429973
GEIS 51 (Palbosarc)	Phase II multicenter trial of palbociclib in second line of advanced sarcomas with CDK4 overexpression	Spain	NCT03242382
GEIS 52 (InmunoSARC)	Phase I–II trial of sunitinib plus nivolumab after standard treatment in advanced soft tissue and bone sarcomas	Spain, Italy	NCT03277924
Sarcome13/0S2016	Randomised phase-2 trial of mifamurtide (MEPACT^®^) combined with post-operative chemotherapy for newly diagnosed patients up to 50 years with high risk osteosarcoma (metastatic or localized disease with poor histologic response to neoadjuvant chemotherapy).	France	NA
Regobone	A phase II study evaluating efficacy and safety of regorafenib in patients with metastatic bone sarcomas	France	NCT02389244
Cabone	Cabozantinib-s-malate in treating patients with relapsed osteosarcoma or Ewing sarcoma	France	NCT02243605
HOPE ITCC-035	Study of lenvatinib in children and adolescents with refractory or relapsed solid malignancies and young adults with osteosarcoma	France, USA, Germany, Italy, Spain, UK	NCT02432274
PembroSARC	Combination of MK3475 and metronomic cyclophosphamide in patients with advanced sarcomas: multicentre phase II trial	France	NCT02406781
PROMO	A phase II study of pembrolizumab in patients with relapsed or metastatic osteosarcoma not eligible for curative surgery	Norway, Italy	NCT03013127
MAPPYACTS	Proof-of-concept study to stratify targeted therapies adapted to molecular profiling	France, Ireland, Israel, Italy, Spain	NCT02613962
ESMART	European proof-of-concept therapeutic stratification trial of molecular anomalies in relapsed or refractory tumors	France, Germany, Italy, Netherlands, Spain, UK	NCT02813135
Can cancer cells be found in blood samples from patients with bone sarcoma?	Enumeration of circulating tumour cells in patients with bone sarcomas: an observational study	UK	ISRCTN29619083

*NA* not available

Nathalie Gaspar, on behalf of The French bone sarcoma group presented the upcoming French Sarcome13/0S2016 trial. This is a first-line randomised Phase-2 trial of mifamurtide (MEPACT^®^) combined with post-operative chemotherapy for newly diagnosed patients up to the age of 50 years with high risk osteosarcoma (metastatic or localized disease with poor histologic response to neoadjuvant chemotherapy). Sarcome13/OS2016 backbone chemotherapy is based on the previous, Unicancer sponsored, French OS2006/Sarcome9 trial (M-EI in children, adolescent and young adults and API-AI in adults) [5, 6]. Post-operative mifamurtide administration for 36 weeks, is randomised in addition to post-operative chemotherapy. This randomized trial, the first since the controversial INT-033 trial, is using a Bayesian design to determine whether the macrophage modulator, mifamurtide, provides benefit to this group of patients [7]. Several phase-2 trials for relapsed osteosarcoma patients are on-going in France testing different multi-tyrosine kinase inhibitors alone, either randomized versus placebo (Regobone from 10 years old), or in single arm phase-2 trial (Cabone from 12 years old), as well as associated with chemotherapy (HOPE, ITCC-035, lenvatinib ± etoposide/ifosfamide up to 25 years old) [8–10]. Recent international studies investigating immune check point inhibitors (anti-PD1 and anti-PDL1), alone have not been as promising as hoped and further work is required to understand mechanisms of resistance and which patients are likely to respond to these agents [11, 12]. Results of a combination French study with metronomic cyclophosphamide are awaited (PembroSARC) [13]. The next trial for paediatric and adult patients with osteosarcoma relapse is being discussed within the ITCC Consortium (Innovative Therapies for Children with Cancer) and consideration is being given to combination of two promising approaches, a multi-tyrosine kinase inhibitor and an immune modulator. Inclusion of osteosarcoma with molecular profiling in the multiarm phase-1/2 trial AcSé-eSMART ITCC-057 provides an opportunity to investigate the activity of a PARP inhibitor in combination with irinotecan or WEE1 inhibitor with carboplatin [14].

Elisa Tirtei, Turin, on behalf of The Italian Sarcoma Group presented a study that opened in November 2016 and is a collaboration with the Italian Onco-Haematology Paediatric Association (AIEOP) and Italian Institute for Genomic Medicine of Turin. It is a prospective, multicentre study to analyse the tumour genomic profile of paediatric and adult patients with new diagnoses of sarcoma or relapsed/refractory sarcoma. By comparing tumour samples with the corresponding non-tumour tissue (e.g. peripheral blood), the aim of the study is to define the genomic profile of each sample taking advantage of a next generation sequencing (NGS) platform. A panel of 410 genes to analyse using the Illumina technique have been chosen. Furthermore, starting from fresh sarcoma samples obtained from surgical biopsies, the group aim to establish in vitro cultures and in vivo patient-derived xenografts (PDX) to generate working models. To date, the study has recruited nine patients and has demonstrated the feasibility of this approach. It will now expand to all paediatric Italian oncology departments, so all patients could benefit from precision medicine and it will be an opportunity to widen preclinical research studies about sarcomas.

The UK National Cancer Research Institute (NCRI) Bone Sarcoma Clinical Studies Group aims to develop UK National Institute Health Research (NIHR) portfolio studies in primary bone tumours. Sandra Strauss, London, presented two studies in osteosarcoma, the first, is a prospective cohort study of patients with newly diagnosed osteosarcoma that aims to (i) describe the experience, treatment and outcomes of patients of all ages in the UK; (ii) to investigate the impact of tumour heterogeneity and tumour evolution on patient outcome through the collection of biological samples; (iii) to validate biomarkers to accelerate selection of patients with high risk disease into Phase Ib/II clinical trials; (iv) to identify factors influencing decisions about local therapy and outcomes. She also outlined a multicenter observational study lead by Kenny Rankin, Newcastle evaluating the potential of using *MT1*-*MMP1* to isolate circulating tumour cells (CTCs) in patients with osteosarcoma at diagnosis and subsequent treatment stages. To date the study has recruited 30 patients across four sites [15].

The session concluded with a presentation by Jakob Anninga, Nijmegen, of a study investigating the effect of dose-intensity on event-free survival (EFS) in groups of patients with localized osteosarcoma. Clinical data from MRC BO06 was used to perform a landmark analysis, which aimed to cluster patients on the similarity of the individual received dose-intensity (iRDI). The group demonstrated a more accurate relationship between iRDI and EFS when investigated by the whole individual treatment-history, i.e. using longitudinal treatment-data, compared to using either intention-to-treat analysis or just the final value of iRDI. Their results suggest that individual tolerability is an important issue in EFS and that maintenance of treatment intensification towards an individual’s biological tolerance is beneficial.

## Genetic targets in osteosarcoma

A number of presentations focused on recent data generated from sequence analyses performed in osteosarcoma, with identification of potential genetic targets and a discussion of the clinical implications of these findings. Sam Behjati, Cambridge, presented findings of the osteosarcoma study of the *International Cancer Genome Consortium*. In that analysis, 112 childhood and adult tumours, encompassing all major histological subtypes, were studied by whole exome (n = 75) or whole genome sequencing (n = 37). In 32/112 cases an actionable mutation was identified, including 20 cases with amplifications of receptor tyrosine kinases. Amongst these were amplifications of *IGF1R* that occurred in ~ 10% of cases, a finding that was validated by FISH in an extension cohort of 87 cases. Overall the findings of this study provide a rationale for a basket trial in osteosarcoma that focuses on actionable mutations, including amplifications of the *IGF1R* gene [16].

Daniel Baumhoer, Basel, presented the findings of a whole exome sequence analysis of 31 osteosarcomas, which demonstrated that these tumours show highly complex karyotypes with abundant structural and numerical aberrations and a multitude of different driver genes. However, despite the great amount of inter- and intratumoural heterogeneity, in their analysis, the majority of tumours (> 85%) appear to acquire a deficiency in homologous recombination (HR) repair that could potentially be therapeutically exploited by using PARP-inhibitors [17]. It is well recognized that PARP-inhibitors are effective in tumours harbouring deleterious germline or somatic mutation in BRCA1/2, i.e. breast and ovarian cancer, as shown by many studies [18]. Patients with wild-type BRCA who have a homologous recombination deficiency (“BRCAness”) have also been shown to be sensitive to PARP inhibition [19] as well as platinum-based chemotherapy [20]. He presented pre-clinical data on the efficacy of PARP-inhibitors in reducing cell viability osteosarcoma cell lines, which has been demonstrated by a number of groups [21]. He concluded on the basis of this evidence, that although there is no universally accepted test to assess the sensitivity of tumours to this treatment (so-called “BRCAness”) in patients upfront, there is a molecular rationale to try this approach also in patients ideally within the framework of a clinical study.

Accurate methods to detect HR-deficiency in clinical samples to identify the patients that can benefit from these treatments are highly sought and a number are under development. Andrea Degasperi, Cambridge, presented a novel method to predict BRCA1 and BRCA2 deficiency based on mutational signatures, called HRDetect, which is a classifier that identifies tumours with a specific type of HR defect—BRCA1 or BRCA2 deficiency in particular [22]. HRDetect was trained on whole genome sequenced (WGS) breast cancers and validated on independent cohorts of breast, ovarian and pancreatic cancers. This classifier is currently the most accurate method for predicting BRCA1 or BRCA2 deficiency in such tumours (AUC ~ 0.98) [22]. However, when applied to osteosarcomas in the ICGC data set, HRDetect predicted that only one sample of the 37 WGS samples tested was similar to BRCA1/BRCA2-deficient breast cancers. Although HRDetect has not yet been validated in osteosarcoma, due to the rarity of BRCA1/BRCA2 deficient bone tumours, this result indicates that BRCA1/BRCA2 deficient-like tumours may not be very common in osteosarcoma and need to be carefully stratified for appropriate treatment. At the same time, other types of HR deficiency may be present in osteosarcoma, that do not resemble BRCA1/BRCA2 deficiency in breast cancer. These results indicate that distinguishing patients with the various forms of genomic instability is not only possible, it is critical.

Ola Myklebost, Oslo, presented studies of the sensitivity of the EuroBoNet osteosarcoma cell line panel to the PARP inhibitor, Talazoparib [23]. The cell lines had variable responses, from similar to that of the highly sensitive BRCA-mutated breast cancer cell line SUM149 to that of the insensitive control HeLa, and responses of the osteosarcoma lines were long-lasting when the drug was removed. Various biomarkers were investigated, including mutations in HRR genes, presence of SNV mutation profile 3, and the sensitivity to platinum drugs. None of these were strongly predictive, but the response to oxaliplatin performed best, with the response being confirmed in vivo in a mouse PDX model. A trial was proposed to investigate predictive markers, including the potential of the highly variable whole genome profiles.

Michaela Nathrath, Munich, representative of the Cooperative Osteosarcoma Study Group’s biology panel, also summarised the case for studying PARP inhibitors in osteosarcoma based on the above evidence of Kovac et al. [17] and data from cell lines suggesting that BRCA-like phenotype is a unifying trait in osteosarcoma. She suggested that the synthetic lethality concept using this BRCAness might be a new effective therapeutic approach in osteosarcoma but also discussed that optimal techniques to select the patients who are likely to respond to PARP inhibitors have still to be defined and also that understanding drug resistance to PARP inhibitors is important. Despite these challenges, a multi-center Phase-II study with a PARP inhibitor combined with chemotherapy including biomarker positive relapsed osteosarcoma patients is planned in Heidelberg/Germany.

The session concluded with a discussion on the evidence presented for the potential role for PARP inhibitors in osteosarcoma. It was agreed that a number of questions remain outstanding, including the challenges of conducting a clinical trial without an optimal biomarker; that PARP inhibitors are most likely to be of benefit in combination with cytotoxic agents but that the optimal combination is presently unknown. It was also felt the osteosarcoma community may not have exploited the value of tyrosine kinase inhibitors (TKIs) in osteosarcoma yet and that benefit in selected patients may have been hidden in previous studies. Discussions covered whether the recent data from Sam Behjati was sufficient to consider further biomarker-driven studies using appropriate TKIs.

## Immunotherapy rationale, targets and other studies

S. Rubina Baglio, Amsterdam, discussed the results of her investigation on the local inflammation of the tumour microenvironment induced by osteosarcoma tumour cells. In a xenograft mouse model of osteosarcoma, the research group demonstrated that the tumour cells release extracellular vesicles (EVs), carrying a membrane-bound form of TGFβ, that “educate” mesenchymal stem cells to support tumour growth and lung metastasis formation. This effect was caused by a switch in the mesenchymal stem cell cytokine expression profile, and could be fully revoked by the administration of the IL-6 receptor antibody tocilizumab [24]. These findings provide a rationale for novel therapeutic strategies based on immunomodulatory drugs for osteosarcoma patients. In addition, the research group found evidence of increased levels of EV-bound TGFβ in the circulation of osteosarcoma patients, which might provide an option for minimally-invasive therapy response monitoring with liquid biopsies. She concluded that the tumour ‘educated’ mesenchymal stem cells might modulate a local immunosuppressive niche, which could be targeted to stop tumour progression.

Kjetil Boye, Oslo, presented the PROMO-study, which commenced in May 2017, and will be carried out in collaboration with Bologna, Italy. This is a non-randomized, investigator-initiated phase-II study with pembrolizumab monotherapy for patients with metastatic, unresectable osteosarcoma. The study has a 2-stage design, and the primary endpoint is clinical benefit rate (SD+PR+CR) at 18 weeks using RECIST v1.1. Secondary endpoints include progression-free survival (PFS), overall response rate and duration of response evaluated by RECIST and immune-related response criteria, response by ^18^F-FDG PET/CT, overall survival, safety and health-related quality of life. A total of 25 patients are expected to be included in the study.

Anne-Marie Cleton-Jansen, Leiden, presented a study of the expression of HLA and PD-L1 as well as the presence of infiltrating lymphocytes in a series of 87 primary osteosarcomas and metastases from 26 patients, to establish whether immunotherapy could be beneficial to metastatic and chemotherapy refractory osteosarcoma. T cell infiltrate and PD-L1 expression increased during disease progression, suggesting that T-cell based immunotherapy with adoptive cell transfer, peptide vaccines or checkpoint blockade could be a suitable approach for metastatic osteosarcoma patients. Down regulation of HLA-A molecules may be a limiting factor, but only in a small fraction of the patients [25].

Elisa Tirtei, Turin, presented the outline of a phase-I trial for the infusion of autologous, cytokine induced killer (CIK) cells in patients with advanced and/or refractory sarcoma. These cells were ex vivo expanded, HLA-unrestricted T-/NK-cells, with NKG2D and MIC A/B as anti-tumour targets [26, 27]. In vitro studies in mice have shown significant tumour cell killing using this method.

The results of a French study to characterize the micro-environment in the biopsies of 126 patients was presented by Francoise Rédini, Nantes. The French phase 3 trial (OS 2006) testing the combination of Zometa^®^ with chemotherapy and surgery did not improve the outcome of patients with osteosarcoma. The authors studied the presence of infiltrating immune cells (CD68/CD163 tumour-infiltrating macrophages, CD8 lymphocytes, osteoclasts, and the PD1/PDL-1 checkpoint) in the biopsies of patients who participated in the OS 2006 trial. It was shown that high CD163 levels significantly correlated with greater overall survival and with longer metastasis PFS independently of diagnosis status. CD8 staining was positive in > 50% of cases with a median staining of 1%. Lower CD8 levels were associated with metastatic disease at diagnosis and only the presence of CD8-positive cells significantly correlated with improved overall survival in patients who were treated with the bisphosphonate Zometa^®^. It was concluded that immunohistochemical analysis of the microenvironment in osteosarcoma patient biopsies could represent a novel tool for therapeutic stratification.

Katia Scotlandi, Italy, presented the results of a study of the effect of Trabectedin on the differentiation and immune environment in a murine osteosarcoma model. Of the two models, mOS13 formed smaller tumours, had an increased bone matrix deposition and a lower metastatic deposition than mOS69 [28]. In the more differentiated tumours a higher monocyte/macrophage (CD68^+^), leucocyte (CD45^+^) and lymphocyte (CD4^+^/CD8^+^) infiltration was demonstrated. Results in patient samples showed a better survival in subjects that had high levels of cytotoxic T-cells (CD8^+^/Tia1^+^: long term cumulative survival 81%, versus 45% in patients who were CD8^−^/Tia^−^) [29]. PD-L1 was positive in 14% of the patients, and had a worse survival in a subset with a CD8^+^ infiltrate. In murine osteosarcoma, Trabectedin inhibited tumour growth and metastasis formation, affected gene-transcription via RUNX2 activation to a more differentiated phenotype, and also induced the recruitment/expansion of adaptive T cells. However, analysis of tumour-infiltrating T cell phenotype and activation state from trabectedin-treated mice showed increased PD-1 checkpoint inhibitor expression on CD8 T cells, compatible with their impaired function. It was concluded from this study that Trabectedin reprogrammed the tumour associated micro-environment, providing the foundation for combination therapies with immune checkpoint inhibitors.

Bone sarcomas and especially osteosarcomas are highly heterogeneous. This heterogeneity both between tumours (inter-tumour heterogeneity) and within tumours (intra-tumour heterogeneity) can be related to genetic and non-genetic factors and introduces significant challenges for classifying patients that might benefit from targeted therapies. It has been suggested that CTCs may reflect the biological evolution (e.g. new mutation events) of primary tumours and associated metastases. Unfortunately, in contrast to carcinomas, in which CTCs have been isolated from epithelial markers (e.g. EpCAM), there are no specific markers expressed by sarcoma cells. However, sarcoma cells like other cancer cells frequently show a differential size compared to normal cells and a lower deformability. Size and deformability criteria have been used in pre-clinical models for isolating CTCs and could serve as proof-of-concept for pilot clinical trials in bone sarcoma [30]. Dominique Heymann, Sheffield, presented a pilot study that is using the Parsortix™ System to enrich for CTCs, which are then isolated and captured using the DEPArray™ System.

Finally, Michel Vanden Eynden, Belgium, presented a project investigating telomere maintenance in paediatric tumours, with a focus on osteosarcoma. Most cancers (85–90%) are known to reactivate telomerase to achieve cellular immortalisation [31]. However, another mechanism, based on homologous recombination between telomeric sequences, called alternative lengthening of Telomeres (ALT), is also able to provide cells with indefinite replication potential. It has been shown that ALT^+^ cells are characterized by the presence of a more relaxed telomeric chromatin that may account for the elevated rates of telomeric sister chromatid recombinations typically found in these cells [32, 33]. ALT is known to be found mainly in paediatric tumours, with about 50% of osteosarcoma showing ALT features [34]. At present, understanding ALT mechanism is a major challenge; as it is absent from normal somatic cells, it represents an attractive target for cancer therapy, but specific inhibitors have not been identified yet. To better understand this mechanism and assess its relevance as a new prognostic biomarker or a new target for therapy, a collaboration with all paediatric oncology departments of university hospitals across Belgium has been created, which will allow tumour collection all across Belgium and further investigation on ALT in paediatric cancers.

## Future networking

The meeting concluded with a discussion on role of future networks for osteosarcoma and commitment to support networking opportunities as being integral to facilitate collaboration and thereby improve outcomes for patients. Opportunities for this lie within newly-developed European Reference Networks (EURACAN/PaedCan), which aim to increase access of patients to specialist care and are currently focusing on developing clinical practice guidelines, training dissemination and determining how shared data collection could benefit improvements in care for rare diseases [35]. It was also noted that SIOP Europe was in advanced planning of a clinical forum bringing together specialists from all tumour types affecting children and young people to allow cross cutting interdisciplinary work. This first Annual Meeting of the European Society for Paediatric Oncology, to be held May 20–25, 2019 in Prague, Czech Republic, is likely to represent a good opportunity to sustain collaborative working between bone tumour experts.

## Conclusion

Osteosarcoma is a challenging disease with little improvement in survival for three decades. To further improve outcomes, international collaboration propelled by initiatives like the European Bone Sarcoma Network is essential. Novel insights from studies on genomics, altered signalling pathways and sarcoma immunology as well as biomarkers to identify patients for clinical trials and to monitor disease were discussed. These built on presentations from previous meetings and included discussion about potential clinical trials, which now need to be taken forward to develop proposals for national and for collaborative research and trials.

## Abbreviations

AcSé-eSMART: Secured access to innovative therapies—European proof-of-concept therapeutic stratification trial of molecular anomalies in relapsed of refractory tumours in children
anti-PD1: anti-Programmed Death 1
anti-PDL1: anti-Programmed Death Ligand 1
API-AI: adriamycin, cisplatin, ifosfamide–adriamycin, ifosfamide
AUC: area under curve
BRCA1/2: BReast CAncer gene 1/2
CDK4: cyclin-dependent kinase 4
CTC: circulating tumour cells
EEC: EURO EWING Consortium
EOI: European Osteosarcoma Intergroup
EURAMOS: EURopean and American Osteosarcoma Study Group
EuroBoNeT: European network to promote research into uncommon cancers in adults and children: pathology, biology and genetics of bone tumours
euroSARC: clinical trials in rare SARComas initiative
FISH: fluorescence in situ hybridization
GEIS: Grupo Espanol de Investigacion en Sarcomas Asociacion
HR: homologous recombination
IGF1R: insulin like growth factor 1 receptor
iRDI: individual received dose intensity
ITCC: innovative therapies for children with cancer
M-EI: methotrexate–etoposide, ifosfamide
MT1-MP1: membrane-type matrix metalloproteinase-1
NCRI: National Cancer Research Institute
NGS: next generation sequencing
NIHR: National Institute for Health Research
OS: overall survival
PARP: poly ADP ribose polymerase
PDX: patient-derived xenograft
PFS: progression-free survival
RRM1: Ribonucleotide Reductase catalytic subunit 1
SFCE: Société Française des Cancers de l’Enfant
SNV: single-nucleotide variant
TKI: tyrosine kinase inhibitor
WEE1: WEE1 G2 Checkpoint Kinase
WGS: whole genome sequencing
